# EHMTI-0124. Central vestibular system modulation in vestibular migraine - a VBM study

**DOI:** 10.1186/1129-2377-15-S1-E42

**Published:** 2014-09-18

**Authors:** S Wurthmann, S Naegel, B Schulte Steinberg, N Theysohn, HC Diener, M Obermann

**Affiliations:** 1 Neurology, University Hospital Essen, Essen, Germany; 2 Diagnostic and Interventional Radiology and Neuroradiology, University Hospital Essen, Essen, Germany

## Introduction

Vestibular migraine affects 1% of the general population and 30-50% of all migraine patients describe occasionally associated vertigo or dizziness.

## Aims

We aimed to identify brain regions altered in vestibular migraine in order to evaluate the connection between migraine and the vestibular system.

## Methods

Seventeen patients with definite vestibular migraine were compared to 17 controls using magnetic resonance imaging based voxel-based-morphometry.

## Results

We found gray matter volume reduction in the superior, inferior and middle (MT/V5) temporal gyrus as well as in the middle cingulate, dorsolateral prefontal, insula, parietal and occipital cortex. A negative correlation of disease duration and GM volume was observed in areas associated with pain and vestibular processing. Moreover, there was a negative correlation between headache severity and prefrontal cortex volume.

## Conclusions

Alterations identified in vestibular migraine resemble those previously described for migraine, but also extent to areas involved in multisensory vestibular control and central vestibular compensation possibly representing the pathoanatomic connection between migraine and the vestibular system.

No conflict of interest.

